# The benefit of indwelling pleural catheter with ambulatory pneumothorax device and autologous blood patch pleurodesis in lymphangioleiomyomatosis with persistent air leak

**DOI:** 10.1002/rcr2.1143

**Published:** 2023-04-12

**Authors:** Boon Hau Ng, Hsueh Jing Low, Nik Nuratiqah Nik Abeed, Mas Fazlin Mohamad Jailaini, Mohamed Faisal Abdul Hamid, Andrea Ban Yu‐Lin

**Affiliations:** ^1^ Pulmonology Unit, Department of Internal Medicine, Faculty of Medicine Universiti Kebangsaan Malaysia Medical Centre Kuala Lumpur Malaysia; ^2^ Department of Anesthesiology & Intensive Care Unit, Faculty of Medicine Universiti Kebangsaan Malaysia Medical Centre Kuala Lumpur Malaysia

**Keywords:** blood patch pleurodesis, indwelling pleural catheter, lymphangioleiomyomatosis, persistent air leak, pneumothorax

## Abstract

We report a 35‐year‐old woman who presented with dyspnoea and chest pain for 1 week. High‐resolution computed tomography (HRCT) thorax revealed bilateral pneumothoraces with diffuse lung cysts. Bilateral intercostal chest tubes were inserted, and there was a persistent air leak (PAL) bilaterally. We performed an autologous blood patch pleurodesis (ABPP) for the left PAL. For the right PAL, she underwent a successful right video‐assisted thoracic (VATS) surgery, wedge biopsy, and surgical pleurodesis. Histopathology examination confirmed the diagnosis of lymphangioleiomyomatosis (LAM). The left pneumothorax recurred. An indwelling pleural catheter (Rocket® IPC™; Rocket Medical plc; WASHINGTON) was inserted and the patient was discharged after 1 day with an atrium pneumostat (Pneumostat™; Atrium Medical Corporation, Hudson, NH, USA) chest drain valve. The patient was initiated on Sirolimus 2 mg daily. The left PAL resolved at 6 weeks. This case highlights the benefit of IPC with an ambulatory pneumothorax device in a patient with LAM with PAL.

## Graphical abstract

Lymphangioleiomyomatosis (LAM) is a rare cystic lung disease with the progressive formation of numerous small cysts. The course of the disease is complicated by recurrent pneumothoraces. This occurs due to cyst rupture into the pleural space or through alveolar wall disruption resulting in an air leak. Chemical or surgical pleurodesis is performed to prevent a recurrence. Persistent air leak (PAL) can complicate this condition. We report the successful use of IPC with an ambulatory pneumothorax device in a patient with LAM with PAL.

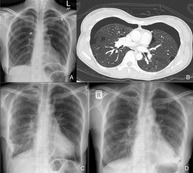

## INTRODUCTION

Lymphangioleiomyomatosis (LAM) is a rare cystic lung disease with the progressive formation of numerous small cysts. The course of the disease is complicated by recurrent pneumothoraces. This occurs due to cyst rupture into the pleural space or through alveolar wall disruption resulting in an air leak. Chemical or surgical pleurodesis is performed to prevent a recurrence. PAL can complicate this condition. We report the successful use of IPC with an ambulatory pneumothorax device in a patient with LAM with PAL.

## CASE REPORT

A 38‐year‐old woman was referred to the respiratory clinic with progressive exertional dyspnoea for 6 months. There was no cough, fever, or weight loss. The respiratory examination was unremarkable. Chest radiograph showed bilateral cystic changes. A working diagnosis of LAM was made. High‐resolution computed tomography (HRCT) thorax revealed diffuse thin‐walled cysts bilaterally. Detailed clinical evaluation including dermatologic and retinal examination did not reveal any evidence suggestive of tuberous sclerosis. Abdominal ultrasonography showed no evidence of associated angiomyolipoma, lymphangioleiomyoma, or lymphadenopathy. The patient declined video‐assisted thoracoscopic surgery (VATS) lung biopsy to confirm the diagnosis.

**FIGURE 1:**
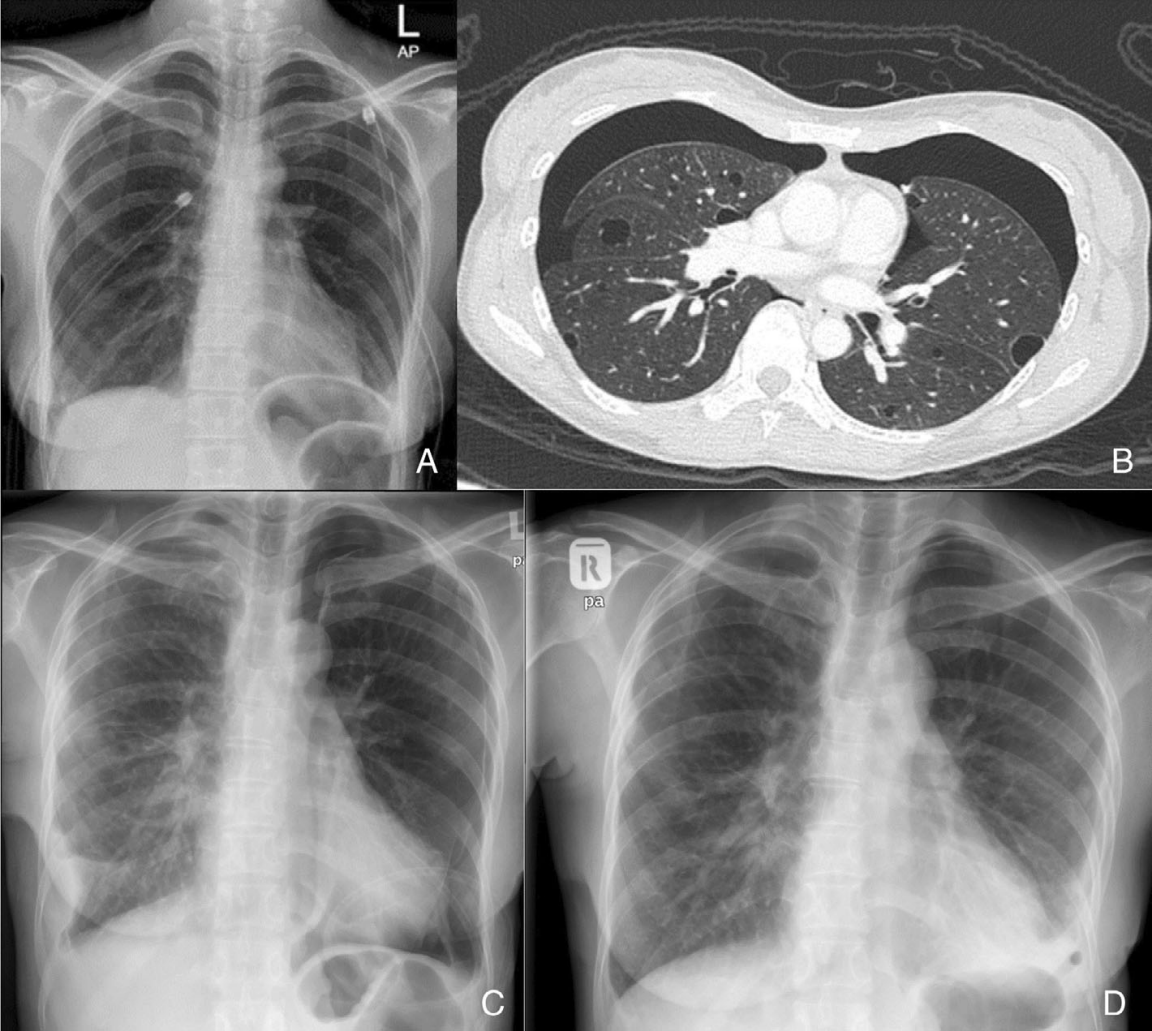
(A) Chest X‐ray showing bilateral pneumothorax with both chest tubes in situ. (B) Computed tomography of the thorax showing pneumothorax and scattered thin‐walled rounded cysts with normal intervening lung parenchyma. (C) Chest radiograph post‐IPC insertion. (D) Chest radiograph 6 weeks after IPC insertion showing resolved PAL and spontaneous autopleurodesis

She presented 6 months later with bilateral pneumothoraces. The oxygen saturation was 94% on room air, respiratory rate of 24 breaths per minute, blood pressure of 110/62 mmHg and heart rate of 96 beats per minute. Respiratory examination revealed bilateral reduced breath sounds. Bilateral chest tubes size of 24 Fr were inserted (Figure 1A). HRCT thorax revealed the progression of diffuse lung cysts with bilateral pneumothorax (Figure 1B).

There was continuous bubbling in both underwater seal devices on day 5 with radiographic evidence of partially expanded lungs bilaterally. The patient was unfit for single‐lung ventilation for the VATS repair of the PAL. We performed an ABPP for the left PAL with the instillation of 2 mL/kg of autologous blood. The left PAL persisted at 72 h requiring a further two ABPP. The left PAL was resolved after 1 week.

After 1 week, the patient underwent a successful right VATS with apical wedge resection, surgical stapling of the right apical lobe, and mechanical pleurodesis to achieve interpleural symphysis. The left PAL recurred 1 day after the VATS for the right PAL. The patient refused further surgical intervention. A decision was made to insert an IPC with an ambulatory pneumothorax device (Figure 1C). She was discharged 1 day after the IPC insertion. Outpatient review at 6 weeks showed complete resolution of the left PAL (Figure 1D). The VATS biopsy confirmed LAM and she was initiated on Sirolimus 2 mg daily. Further reviews at months 3, 6, and 9 showed no recurrence of pneumothorax.

## DISCUSSION

Lymphangioleiomyomatosis (LAM) is a rare cystic lung disease, which can occur sporadically or in association with tuberous sclerosis. LAM predominantly affects females of child‐bearing age. It is characterized by the proliferation of smooth muscle‐like cells throughout the interstitium of the lung and spread via the lymphatic tracts.^1^

The commonest chest radiograph abnormality is reticulonodular shadowing and cysts or bullae with preserved lung volumes.^2^ Computed tomography of the thorax shows multiple thin‐walled cysts scattered throughout the lung fields in an even distribution with normal intervening lung parenchyma. The diffuse thin‐walled cysts, ranges from 2 to 5 mm in diameter but can be as large as 30 mm. They are uniformly round or ovoid, and without preference for upper or lower lung zones.^3^ Lung function commonly shows an airflow obstruction with reduced diffusing capacity for carbon monoxide.^4^

The definitive diagnosis of LAM requires characteristic CT findings and a lung biopsy showing the characteristic histological features of LAM and reactivity with HMB‐45. A biopsy is not needed when there is presence of tuberous sclerosis complex, angiomyolipoma, chylous effusions, lymphangioleiomyoma, or high VEGF‐D value (greater than 800 pg/mL) in a patient with typical CT findings.^5^

The initial approach to pneumothorax in LAM is similar to other lung diseases. Surgical intervention is considered in recurrent pneumothoraces or PAL. The persistence of pneumothorax with PAL beyond 3–5 days requires evaluation for surgery to close the air leak followed by pleurodesis.^6^

Autologous blood patch pleurodesis (ABPP) and endoscopically placed ‘one‐way’ endobronchial valves have demonstrated succes in patients unfit or refuse surgery.^7^

ABPP is comparable in efficacy to talc, tetracycline, or silver nitrate in achieving pleurodesis with less pain, fever and shorter length of hospitalization.^8^ The reported amount of blood used in ABPP varies from 0.5 to 2 mL/kg.^9^ Systemic review has shown the success rate of ABPP to be from 27% to 82%.^9^ In our patient, the PAL responded to ABPP initially but the pneumothorax recurred after intubation for VATS.

The subsequent application of an IPC with the transition to an ambulatory drainage device allowed the leak to heal and reduced the length of hospitalization. The literature review pertaining to the treatment of LAM and pneumothorax is shown below (Table 1). The majority were treated with tube thoracotomy (TT). None were treated with the triple approach of TT, ABPP and IPC.

**TABLE 1:** Literature review of the LAM, site of the pneumothorax and intervention

Author/year	Age/sex	Site	PAL	Intervention
Lamya Al Aamri et al.^11^	31/F	Right	No	TT
Narath Carlile et al.^12^	31/F	Left	Yes	TT + VATS + PD
Thatti et al.^13^	44/F	Left	–	TT + RATS + PD
Odak et al.^14^	24/F	Right	No	TT
Kania et al.^15^	39/F	Left	No	TT + VATS + PD
Vishwanath Pujari et al.^16^	15/M	Bilateral	Yes	TT + PD (doxycycline)
Duarte Lages Silva et al.^17^	35/F	Right	No	TT
Demirci et al.^18^	25/F	Right	No	TT
Bogoni Giuliane et al.^19^	44/F	Bilateral	Yes	TT + PD (Talc)
Jack Amja et al.^20^	35/F	Left	No	TT
Stacey Ho et al.^21^	42/F	Bilateral	No	TT
Verma et al.^22^	27/F	Left	No	TT
Faisal et al.^23^	41/F	Bilateral	Yes	TT + VATS + PD (Talc)
Mahishale Vinay et al.^24^	16/F	Left	No	TT
Restrepo‐Gualterosa et al.^25^	16/F	Left	No	TT
Kazuhiri Wakida et al.^26^	17‐M	Right	No	TT + VATS
Jain et al.^27^	36/F	Right	Yes	TT
Abhishek et al.^28^	36/F	Left	No	Conservative
Baykal et al.^29^	19/F	Right	No	TT
Małgorzata Wojtyś et al.^30^	25/F	Bilateral	Yes	TT + VATS + Left pleurectomy
	33/F	Bilateral	Yes	TT + VATS + Pleurectomy
	44/F	Right	Yes	TT + VATS + PD
	29/F	Left	No	TT + VATS Pleurectomy
	40/F	Bilateral	No	TT + PD
Riojas et al.^31^	28/F	Right	Yes	TT + VATS + PD
Cheng et al.^32^	30/F	Bilateral	–	TT + VATS + PD
Johnston et al.^33^	18/F	Left	Yes	TT + VATS
Berkman et al.^34^	46/F	Bilateral	Yes	TT + Midline sternotomy + Resection of bullae

Abbreviations: L, left; PD, pleurodesis; R, right; RATS, robot‐assisted thoracic surgery; TS, tuberous sclerosis; TT, tube thoracotomy; VATS, video‐assisted thoracic surgery.

Treatment with Sirolimus stabilizes pulmonary function, prevents pneumothorax recurrence, improves oxygenation, exercise capability, and quality of life in LAM.^10^ Sirolimus is indicated in patients with moderate to severe lung function impairment, which is defined by an FEV1 < 70% predicted. Patients with disease progression on sirolimus or progressive disease with FEV1 < 30% predicted and those dependent on oxygen should be referred for evaluation for a lung transplant.

PAL in secondary pneumothorax is a clinical challenge as many patients are unfit for surgical intervention. Blood patch pleurodesis or IPC with an ambulatory pneumothorax device is a useful and safe alternative option for these patients.

## CONFLICT OF INTEREST STATEMENT

Andrea Ban Yu‐Lin is an Editorial Board member of Respirology Case Reports and a co‐author of this article. They were excluded from all editorial decision‐making related to the acceptance of this article for publication. Andrea Ban Yu‐Lin is an Associate Editor for the Journal. The other authors have no conflict of interest to declare.

## ETHICS STATEMENT

The authors declare that appropriate written informed consent was obtained for publication of this case report and accompanying images.

## Data Availability

Author elects to not share data
